# Research on non-destructive detection model of tomato fruit quality based on electrical properties and machine learning algorithms

**DOI:** 10.3389/fpls.2025.1690652

**Published:** 2025-10-29

**Authors:** Tingting Wang, Zhanming Tan, Yunxia Cheng, Xinchao Ma, Yongming Wang

**Affiliations:** ^1^ Key Laboratory of Southern Xinjiang Production and Construction Corps, College of Horticulture and Forestry, Tarim University, Alar, Xinjiang, China; ^2^ Facility Agriculture Department, First Division Agricultural Science Research Institute, Alar, Xinjiang, China

**Keywords:** tomatoes, electrical parameters, fruit quality, LSTMAE-XGBoost, non-destructive detection

## Abstract

To break through the limitations of traditional destructive detection methods, achieve rapid, non-destructive, and accurate detection of internal tomato quality, and provide more efficient technical means for agricultural product quality assessment, this study proposes a novel predictive method that integrates a Long Short-Term Memory Autoencoder (LSTMAE) and XGBoost (LSTMAE–XGBoost). This method combines the feature extraction capabilities of the autoencoder, the sequence data processing abilities of LSTM, and the high-precision prediction capabilities of XGBoost. Within the frequency range of 0.1–1000 kHz, electrical parameters such as parallel equivalent capacitance, parallel equivalent resistance, and quality factor—among nine electrical parameters—were obtained from 300 tomato samples using an electrical parameter analyzer. Additionally, four indicators—vitamin C, soluble sugar, soluble protein, and titratable acidity—were obtained through physicochemical analysis of the tomatoes. Based on the electrical parameters and internal physicochemical indicator data of the tomatoes, a non-destructive detection model for tomato internal quality indicators was constructed. Experimental results demonstrate that the LSTMAE–XGBoost model exhibits superior adaptability. In the test set, the coefficients of determination for vitamin C, soluble sugar, soluble protein, and titratable acidity were 0.805, 0.945, 0.838, and 0.845, respectively. Compared to traditional machine learning models, this model offers better prediction accuracy. It improves upon the traditional Pearson correlation coefficient (PCC) feature extraction method by 14.3%, 13.1%, 7.8%, and 9.5%, respectively. Furthermore, LSTMAE–XGBoost can simultaneously predict all four indicators, enhancing the model’s efficiency. Therefore, LSTMAE–XGBoost can be utilized as an effective ensemble model for non-destructive detection of tomato internal quality indicators, which holds significant importance for fruit quality non destructive detection in the horticultural industry.

## 1 Introduction

The tomato (*Solanum lycopersicum* L.), native to South America, is an essential horticultural crop (El-Bendary et al., 2015). The United States, Italy, and China are the primary producers, making it one of the most widely cultivated fruit vegetables globally (Li et al., 2023). According to data from the Food and Agriculture Organization of the United Nations (FAO), in 2022, tomatoes were cultivated in 170 countries worldwide, with a fresh fruit production totaling approximately 186.2 million metric tons (Food and Agriculture Organization of the United Nations (FAO), 2021). As living standards improve, consumers increasingly demand higher nutritional quality in tomatoes (Kyriacou and Rouphael, 2018). The internal physicochemical indices of tomato fruits are crucial determinants of their quality and flavor (Saliba-Colombani et al., 2001), with the sugar-acid ratio influencing taste and the contents of soluble proteins and vitamin C reflecting their nutritional value (Gao et al., 2023). Therefore, fruit quality inspection techniques and assessments are pivotal in advancing the industry’s development, playing a significant role in the grading and classification of tomatoes and aiding consumers in making informed choices. Traditional methods for assessing tomato fruit quality, such as chemical analysis, are destructive, inefficient, and costly. Consequently, there is an urgent need for non-destructive detection techniques that can rapidly and accurately evaluate tomato fruit quality.

In recent years, non-destructive detection techniques have gained widespread application in the agricultural sector. Numerous non-destructive testing (NDT) methods for fruits and vegetables exist, based on physical properties such as light, sound, electricity, heat, and magnetism. These methods include spectroscopic NDT, ultrasonic NDT, and electrical properties NDT, as well as techniques based on fruit aromas and surface conditions, such as electronic nose technology and machine vision (Abasi et al., 2018; Zhang W. et al., 2018; Nouri and Mehdizadeh, 2024). Optical techniques mainly employ Fourier Transform Infrared (FTIR), Near-Infrared (NIR), Raman spectroscopy, and Hyperspectral Imaging (HSI) (Si et al., 2022; Aline et al., 2023) to assess the appearance and intrinsic quality indices of fruits and vegetables. However, physical and bio-logical variations in fruits and vegetables can affect light propagation and interaction with incident light, thus reducing the accuracy of internal quality assessments (Zhang B. et al., 2018). Acoustic methods, primarily ultrasonics, are used to assess quality attributes such as ripeness and sugar content non-destructively (Morrison and Abeyratne, 2014), but they are susceptible to environmental noise and vibrations, which can compromise the assessment results (Jie and Wei, 2018). Electronic nose technology is mainly used to analyze aroma, odor, and flavor in fruits and vegetables, using the emitted scents to judge qualities like ripeness (Saevels et al., 2003; Ali et al., 2020). Imaging is primarily used in re-search on fruit ripeness, mechanical damage, and classification (Mahanti et al., 2022; Palumbo et al., 2023). In summary, this study utilizes electrical properties for non-destructive evaluation of tomato fruit internal quality. The acquisition of electrical parameters offers the advantages of rapidness, non-destructiveness, and high efficiency, making it more suitable compared to other methods.

The postharvest storage temperature significantly impacts tomato fruit quality. Studies by X. Li et al (Li et al., 2025). demonstrated that low-temperature conditions (4°C and 14°C) effectively maintain fruit appearance and preserve soluble solid content. To investigate the effects of storage temperature on quality and flavor of ripe red tomatoes, Tao et al (Tao et al., 2024). analyzed transcriptomes and volatile metabolomes of fruits stored at 0°C and 20°C for 8 days. The results showed that storage at 0°C suppressed the expression of pectinesterase (PE) and β-galactosidase (β-GAL), thereby maintaining better fruit texture and color. To examine the effects of low temperature on quality and flavor of ripe red tomatoes, Guan et al (Guan et al., 2025). analyzed volatile organic compounds (VOCs) in fruits stored at 4°C and 25°C using headspace-gas chromatography-ion mobility spectrometry (HS-GC-IMS). The results demonstrated that storage at 4°C better maintained sensory quality, color, firmness, and soluble solids content (SSC) in cherry tomatoes harvested at the red-ripe stage. Accordingly, this study selected two storage temperatures (4°C and ambient) to increase machine learning sample size while facilitating nondestructive and rapid detection of fruit quality changes.

Based on electrical properties and machine learning algorithms, non-destructive detection techniques have been applied to various crops. For instance, Ibba (2021) utilized a constructed variable oscillator platform to measure the postharvest dielectric properties of jujube fruits, and employed SVR and MLP models for moisture prediction and quality classification of jujubes. Zhang et al. (2022) employed electrical parameters and a Generalized Regression Neural Network (GRNN) to develop a non-destructive detection method for firmness during the ripening stage of Xiangli pears. The model exhibited good predictive performance, with an R² value of 0.9628 and an RMSE of 0.383. Yu et al. (2022) utilized electrical parameters and an Adaptive Neuro-Fuzzy Inference System (ANFIS) to develop quality models for firmness, soluble solids content, and color difference (a*) in Xiangli pears. Mellyana et al. (2024) employed electrical properties (impedance, admittance, resistance, and capacitance) within the frequency range of 50 Hz to 5 MHz, along with Multiple Linear Regression (MLR) and Artificial Neural Network (ANN) models, to directly predict the free fatty acid and moisture content in oil palm fruits. Through comparative analysis of the models, ANN exhibited the best performance in predicting both parameters. Lin et al. (2024) developed a platform for analyzing the electrical properties of fruits to detect electrical parameters in passion fruits. They employed Recursive Feature Elimination with Cross-Validation (RFECV), Permutation Importance based on Random Forest Regression (PI-RF), Per-mutation Importance based on Linear Regression (PI-LR), and Genetic Algorithm (GA) for feature extraction. The extracted electrical parameter features were used as inputs, with quality indicators as outputs. Subsequently, they utilized Extreme Gradient Boosting (XGBoost) and Categorical Boosting (Cat-Boost) models to predict indicators such as soluble solids content (SSC), acidity, and pulp content in passion fruits. Karimi, H. et al (Karimi, 2025) developed an intelligent capacitance system integrated with a variable oscillator platform to measure the dielectric properties of postharvest jujube fruits. They utilized Support Vector Regression (SVR) to predict the moisture content of the jujubes, achieving an R² value of 0.88 and an RMSE of 0.094. Additionally, they employed an MLP model to classify the maturity stages of the jujubes, obtaining F1 scores of 0.87, 0.60, and 0.68 for three different maturity stages. Masoumi, F. et al (Masoumi et al., 2024) utilized non-contact instruments and data linear fitting to elucidate the response of electrical properties during the aging process of bananas and nectarines. They clarified the complex relationships among aging, mass loss, damage detection, capacitance reduction, and fruit characteristics, providing an effective means for non-destructive quality assessment of fruits. Although non-destructive detection methods based on electrical parameters have been applied to some fruits and vegetables, most of the machine learning algorithms currently in use are unable to perform automatic feature extraction and require manual design. This significantly limits the modeling process due to potential errors in manual feature extraction and inherent defects of the technology itself (Li et al., 2024). Therefore, it is highly necessary to utilize deep learning to achieve an end-to-end model process.

Based on previous research, this study is the first to systematically apply LSTMAE for feature extraction from tomato electrical parameters, providing a novel and automated feature engineering solution for predicting fruit quality indicators based on electrical parameters. Compared to traditional machine learning models, deep learning adopts an end-to-end learning approach, mapping raw data directly to the final results, thereby simplifying the machine learning process (Taye, 2023). This learning method minimizes manual intervention and enhances the model’s efficiency and accuracy. This model is of significant importance for improving the efficiency of tomato quality detection, reducing costs, and promoting agricultural intelligence.

**Figure 1:**
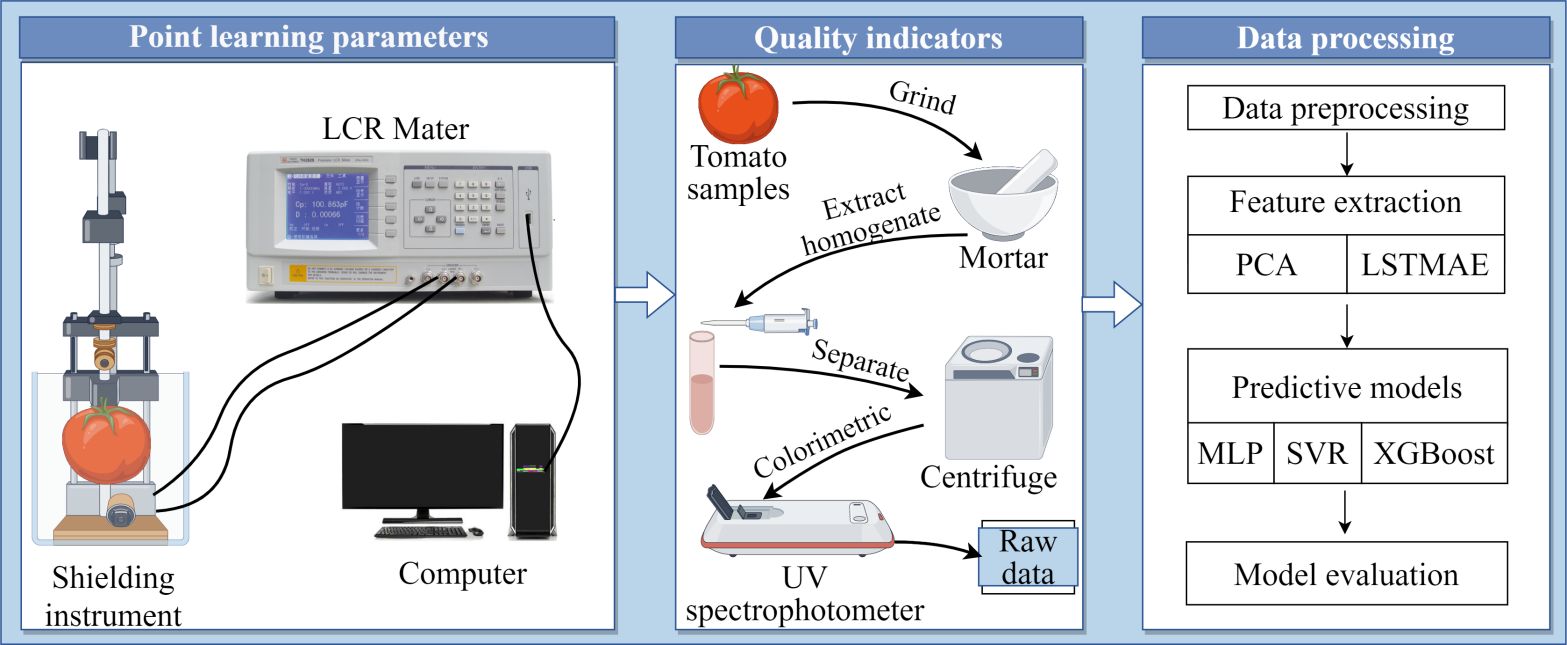
Workflow of the current study.

The main work of this study comprises the following sections: (1) Tomato electrical parameter measurement, where nine electrical parameters of tomato samples were detected using an electrical parameter meter; (2) Tomato quality indicator measurement, where four indicators—vitamin C, soluble sugar, soluble protein, and titratable acidity—of tomato samples were analyzed using chemical methods; (3) Construction of non-destructive testing models, namely “LSTMAE-XGBoost,” “LSTMAE-SVR,” and “LSTMAE-MLP,” using automatic feature extraction methods, with a comparison to non-automatic feature models, namely “PCC-XGBoost,” “PCC-SVR,” and “PCC-MLP,” constructed using Pearson correlation coefficient analysis. The workflow is illustrated in **Figure 1**.

## 2 Materials and methods

### 2.1 Source and treatment of tomato samples

**Figure 2:**
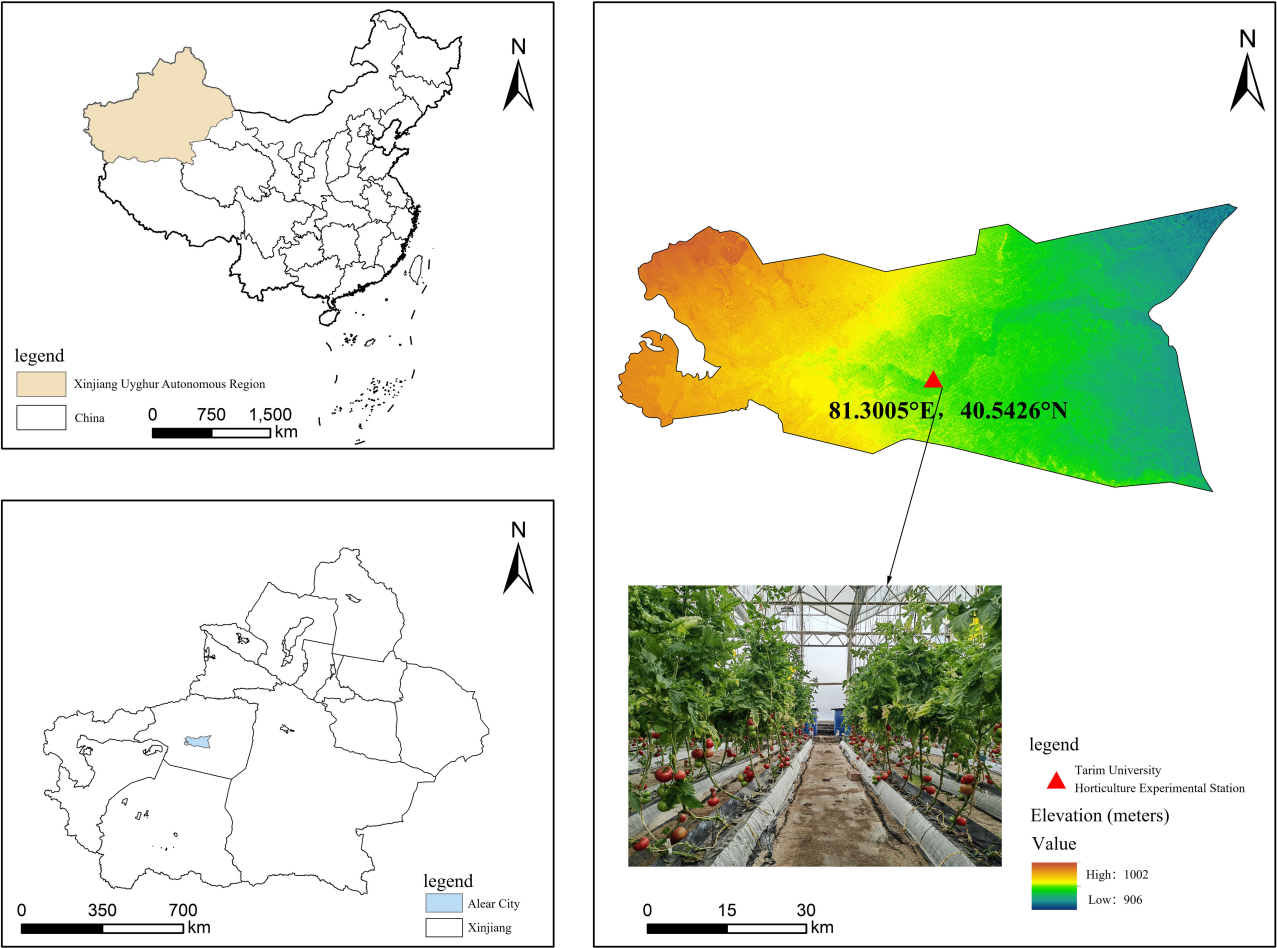
Geographic location of the horticultural experiment station and samples of tomatoes.

Tomato samples were selected from the ‘Dongfeng 199’ (**Figure 2**) variety grown in a contiguous greenhouse at the Horticultural Experiment Station of Tarim University in Aral, situated at 81°29’ E longitude and 40°54’ N latitude. The second truss of fruits, uniform in size and color and free from pests and diseases, was chosen during the red ripe stage, totaling 300 fresh fruit samples. These were transplanted on September 28, 2023, with seedlings at the seven leaf stage into cultivation troughs. The tomatoes were cultivated under controlled environmental conditions with day and night temperatures maintained at 25 ± 2°C and 18 ± 2°C, respectively. The relative humidity was regulated at approximately 60%. A drip irrigation system was utilized to deliver water and essential nutrients directly to the root zone. Harvesting took place on February 1 of the following year. Immediately after harvesting, all tomatoes underwent weighing, grouping, and other experimental operations to provide a solid foundation for subsequent studies.

The samples were divided evenly into two groups: 150 samples were stored at room temperature (25°C), and the other 150 samples were stored at a low temperature (4°C). After storage periods of 0, 7, 14, 21, and 28 days, 30 samples from both the room temperature and low-temperature storage were selected for analysis of electrical parameters and intrinsic quality data. Due to spoilage at room temperature storage on days 21 and 28, a total of 294 data sets were ultimately compiled.

### 2.2 Measurement of electrical parameters and quality indices

#### 2.2.1 Measurement of electrical parameters

**Figure 3:**
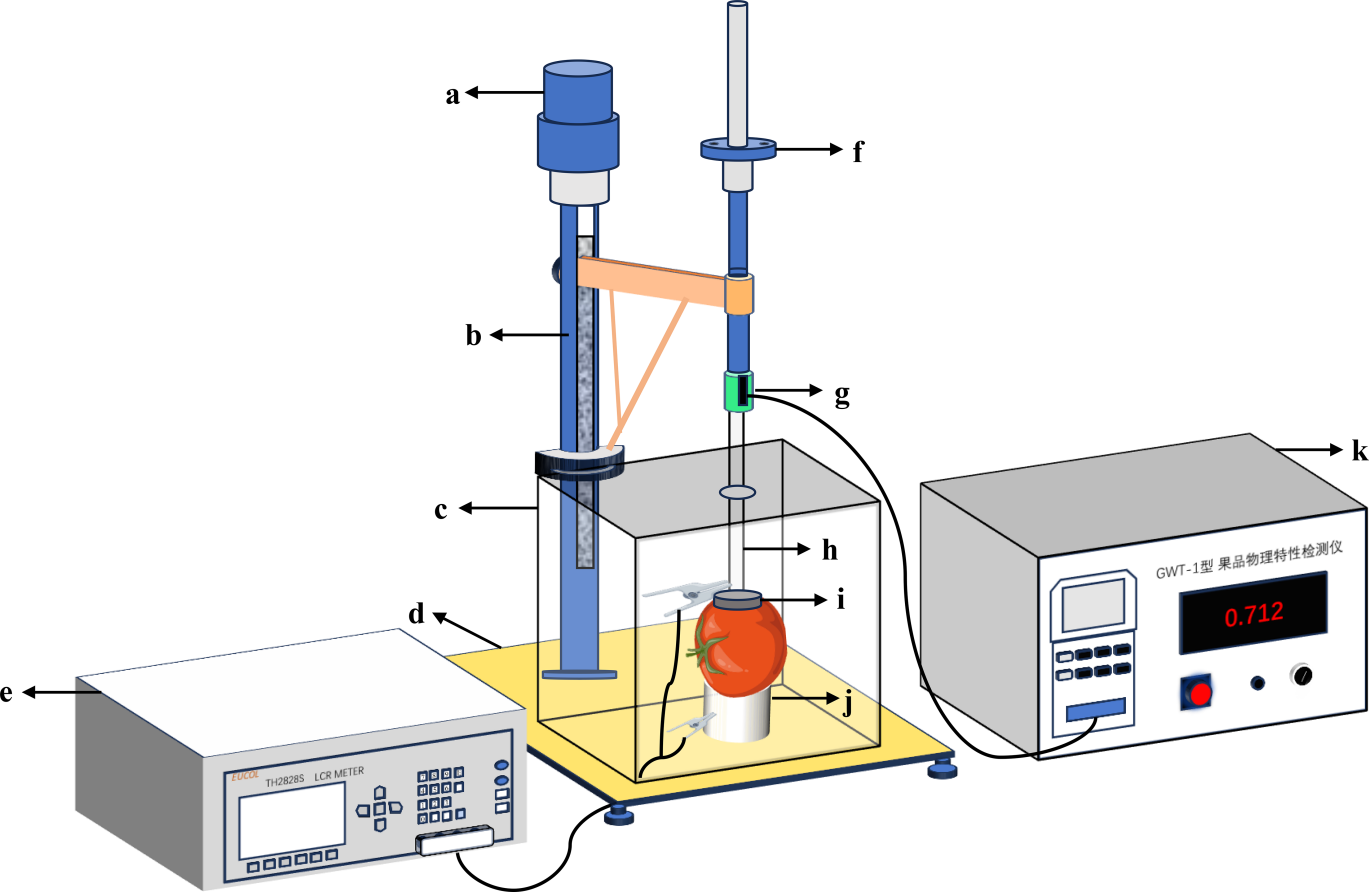
Tomato electrical parameters measurement device. The meaning of the numbers in the picture: **(a)** Loading motor; **(b)** Support frame; **(c)** Shielding box; **(d)** Base; **(e)** Testing bridge; **(f)** Fine-tuning handwheel; **(g)** Force sensor; **(h)** Insulating rod; **(i)** Upper electrode plate; **(j)** Lower electrode plate; **(k)** Force control unit.

Electrical parameters of tomatoes were measured using a custom-built LCR digital bridge analyzer (Zhang et al., 2022) (**Figure 3**). The instrument was preheated for one hour before use and zeroed to minimize errors. Testing frequencies were set at 0.1 kHz, 1 kHz, 10 kHz, 100 kHz, and 1 MHz, with a constant testing voltage of 1V, A pressure of 1N was maintained between the electrode plates. The cheek area of the tomato was clamped with parallel electrode plates to measure nine parameters: parallel equivalent capacitance (Cp), parallel equivalent resistance (Rp), loss factor (D), quality factor (Q), com-plex impedance (Z), resistance (R), conductance (G), inductance (X), and the angular line of complex impedance (θ). These parameters provide critical data for model construction.

#### 2.2.2 Measurement of quality indices

Following the measurement of electrical parameters, the tomatoes were ground into a homogeneous pulp for the determination of key quality indices. Four critical quality indices of tomatoes were analyzed: soluble sugars, titratable acidity, vitamin C, and soluble proteins. Soluble sugar content was determined using the anthrone colorimetric method (Ibba, 2021; Palumbo et al., 2023); soluble protein content was assessed using the Coomassie Brilliant Blue G-250 staining method (Zhang et al., 2022); titratable acidity was measured by acidimetric titration (Yu et al., 2022); and vitamin C was quantified using UV spectrophotometry (Ye et al., 2023). For each measurement, 60 fruit samples were tested in replicate, and the average value was taken as the final result for each sample. The finally measured quality indicator data are shown in **Table 1**.

**Table 1:** Quality indicator data for tomato samples.

Tomato sample data						
Variables	Variable interpretation	Unit	Mean	Maximum value	Minimum value	Std dev
Vc	Vitamin C	mg/100g	15.252	24.448	7.708	2.941
Soluble protein	Soluble protein	mg/g	0.912	2.741	0.100	0.731
soluble sugars	Soluble sugars	%	5.606	9.482	3.311	1.538
TA	Titratable Acids	mmol/100g	0.138	0.401	0.049	0.098

### 2.3 Data preprocessing

To eliminate the influence of different measurement units, all data were normalized to the range [0, 1] using the min-max scaling method, as defined by Equation 1.


(1) $$x^{\prime }=\frac{x-x_{min}}{x_{max}-x_{min}}$$

The electrical parameters were utilized as inputs to the predictive model for forecasting the four tomato quality indicators. Subsequent analysis of the correlations between the parameters and the indicators was conducted. Due to the potential presence of data redundancy and irrelevant variables among the nine electrical parameters, feature extraction and dimensionality reduction were implemented. Specifically, features were extracted through two methods: a manual approach based on the Pearson correlation coefficient and an automated technique employing a Long Short-Term Memory Autoencoder (LSTMAE). The outcomes of the prediction models derived from these feature sets were evaluated and compared.

#### 2.3.1 Feature extraction using pearson correlation coefficient

Feature extraction is performed using the Pearson correlation coefficient, where the value of the correlation coefficient (r) ranges from [-1, 1]. A larger absolute value of (r) indicates a higher degree of correlation, while a correlation coefficient of 0 signifies no correlation. The calculation formula for r is presented in Equation 2.


(2) $$r=\frac{\Sigma_{i=1}^{n}(X_{i}-\bar{X})(Y_{i}-\bar{Y})}{\sqrt{\Sigma_{i=1}^{n}(X_{i}-\bar{X})}\sqrt{\Sigma_{\text{i}=1}^{n}(Y_{i}-\bar{Y})}}$$

Here, $\bar{X\;}$ and $\bar{\;Y}$ denote the sample means of the two variables; $X_{i}$ and $Y_{i}$ represent the ith observation values for each variable, respectively, and n stands for the total number of observations.

#### 2.3.2 Feature extraction using LSTMAE

**Figure 4:**
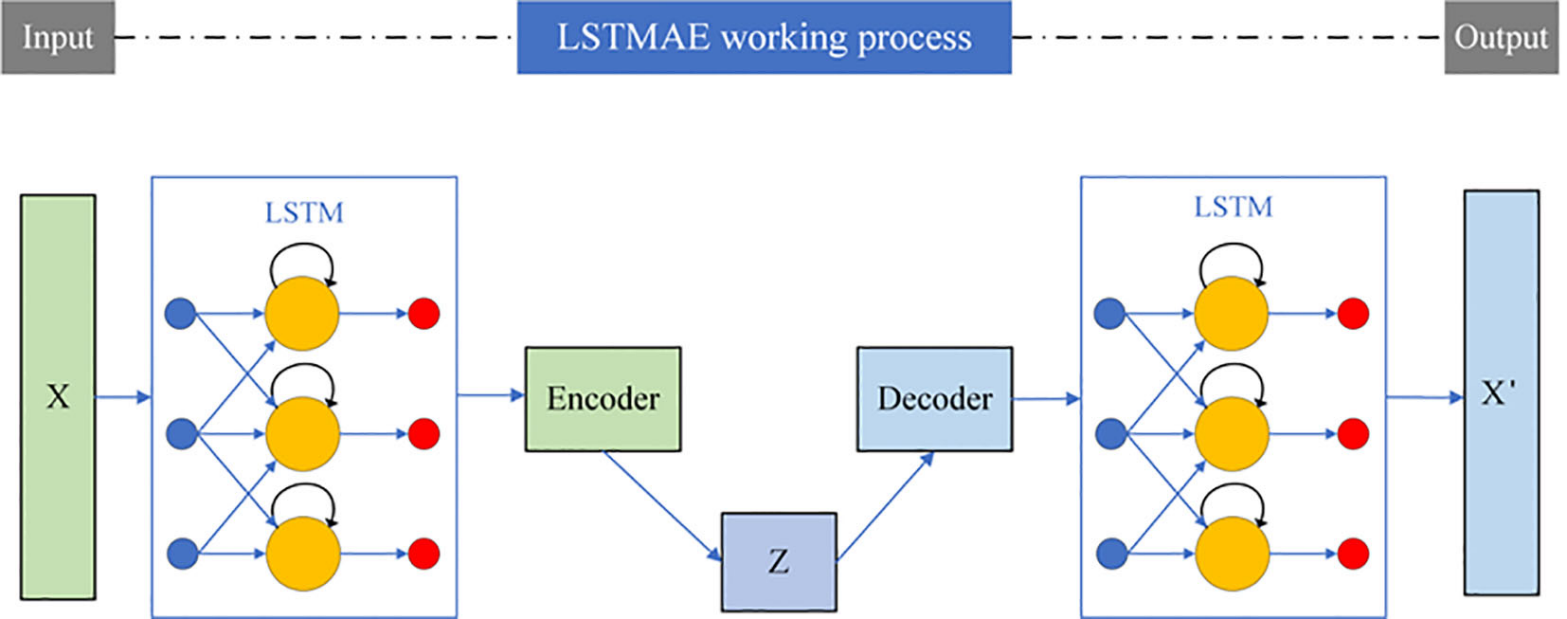
Structure diagram of LSTMAE.

The primary purpose of an encoder is to obtain input data, pass it through a model for reconstruction, and retain a small number of the most important features in the re-constructed input data. The experiments in this paper involve the storage of tomatoes, and two LSTM layers are used before and after the encoding-decoding module of the autoencoder to capture temporal dependencies. Therefore, feature extraction is con-ducted using LSTMAE, and the definition of the LSTMAE model is illustrated in **Figure 4**.

The primary objective of the LSTMAE is to enable the machine to learn and extract the most critical features from time series data. As illustrated in **Figure 4**, the model consists of two main components. The first part is the “encoder”, wherein an LSTM neural network sequentially processes the entire input sequence x and compresses it into a compact, fixed-length “context vector” that captures the essential patterns of the sequence. The core functionality of the LSTM lies in its “gating mechanism”, which regulates the flow of information through three types of gates. The following mathematical formulations describe the computations within a single LSTM unit at time step t.

(a) The forget gate determines which information should be discarded from the cell state, as formulated in Equation 3.


(3) $$f_{t}=\sigma (w_{f}\cdot [h_{t-1},x_{t}]+b_{f})$$

The notation $\left[h_{t-1},x_{t}\right]$ denotes the concatenation of the hidden state $h_{t-1}$ and the current input $x_{t}$ into a longer vector. Here, $w_{f}$ represents the weight matrix of the forget gate, $b_{f}$ denotes the bias term of the forget gate, and σ refers to the Sigmoid activation function.

(b) The input gate regulates which information is stored in the cell state, as mathematically expressed in Equations 4 and 5.


(4) it=σ(Wi·[ht−1,xt]+bi)

(5) $${\vec{c}}_{t}=\text{tanh}(Wc\cdot [ht-1,xt]+bc)$$

The input gate *it* also uses the σ activation function, ${\vec{c}}_{t}$ represents the candidate cell state, and the tanh activation function is used.

(c) The output gate controls which information is propagated to the next time step, as formally defined by Equation 6.


(6) ot =σ(Wo ·[ht−1 ,xt ]+bo)

The output gate *ot*​ determines which information in the cell state ${\vec{c}}_{t}$ will be output.

(d) Upon processing the entire input sequence, the final hidden state and cell state of the encoder collectively form the encoded representation—also referred to as the context vector C—which encapsulates the essential information of the sequence. The mathematical formulation of C is given by Equation 7.


(7) C=(hTenc ,cTenc)

*hTenc* represents the hidden state of the encoder LSTM after processing the last element (T-th) of the entire input sequence, and *cTenc*​ represents the cell state of the encoder LSTM at time step T.

The second component is the decoder, which employs a separate LSTM network to reconstruct the original sequence from the context vector as accurately as possible. The entire model is optimized by minimizing the reconstruction error between the original input sequence and the reconstructed output.

(e) During the decoding phase, the decoder LSTM is initialized with the context vector C, as mathematically defined in Equation 8. It then proceeds to generate the output sequence $\hat{x}$ step by step. The output from the top layer of the decoder is typically passed through a fully connected layer (Dense Layer) to project the LSTM outputs into the desired dimensional space, as formulated in Equation 9.


(8) h0dec=hTenc,c0dec=cTenc

(9) $$x^{\prime }=Woutput\;\cdot htdec\;+boutput$$

The notation *h*0*dec*​=*hTenc* indicates that the initial hidden state of the decoder is set to the final hidden state of the encoder; similarly, *c*0*dec*​=*cTenc* denotes that the initial cell state of the decoder is initialized with the final cell state of the encoder. The term $x^{\prime }$ represents the output of the decoding layer, while *Woutput* and *boutput* correspond to the weight matrix and bias vector of a fully connected (dense) layer, respectively. The variable *htdec* refers to the hidden state generated by the decoder at time step t.

The compression-reconstruction process compels the model to learn and retain the most representative features of the sequence while discarding non-essential noise. As a result, the model demonstrates outstanding performance in tasks such as anomaly detection, data dimensionality reduction, and sequence denoising. Meanwhile, Compared to the PCC method, LSTMAE can directly use the extracted features as input and four prediction indicators as output, enabling the construction of a multi-output prediction model that improves the prediction efficiency of the model.

In this study, both the encoder and decoder were constructed using a single-layer LSTM architecture. The encoder was configured with 3 neurons, whereas the decoder consisted of 10 neurons. The ReLU activation function was employed in both components. The parameter “return_sequences=True” was set to ensure the complete output sequence is returned. The RepeatVector layer was configured with a time step of 5 to align the encoder’s output with the decoder’s input requirements. Furthermore, the TimeDistributed layer was applied with an output dimension of 10, enabling a fully connected operation at each time step. The detailed hyperparameter configurations are provided in **Table 2**.

**Table 2:** The primary parameter configuration for LSTMAE-SVR, LSTMAE-XGBoost, and LSTMAE-MLP.

Parameter settings		
Model name	Key parameters	Parameter settings
LSTMAE	Number of LSTM layers in the encoder	1
	Number of LSTM layers in the decoder	1
	Number of units in encoder LSTM layers	3
	Number of units in decoder LSTM layers	10
	timesteps	5
	features	10
LSTMAE-SVR	Kernel	rbf
	Epsilon(*ϵ*)	0.1
	Regularization parameter (C)	1
LSTMAE-XGBoost	objective	reg:squarederror
	Base Learner	gbtree
	n_estimators	200
	max_depth	7
	min_child_weight	1
	gamma	1
	subsample	0.8
	colsample_bytree	1
	Learning Rate	0.1
LSTMAE-MLP	Number of Hidden Layers	2
	Learning Rate	0.01
	batch_size	32
	epochs	300
	Activation Function	Sigmoid

### 2.4 Model construction

This paper utilizes preprocessed electrical parameters as inputs and tomato quality prediction indices as outputs, employing three nonlinear regression methods for predicting the quality indices: SVR, MLP, and XGBoost.

#### 2.4.1 SVR model

**Figure 5:**
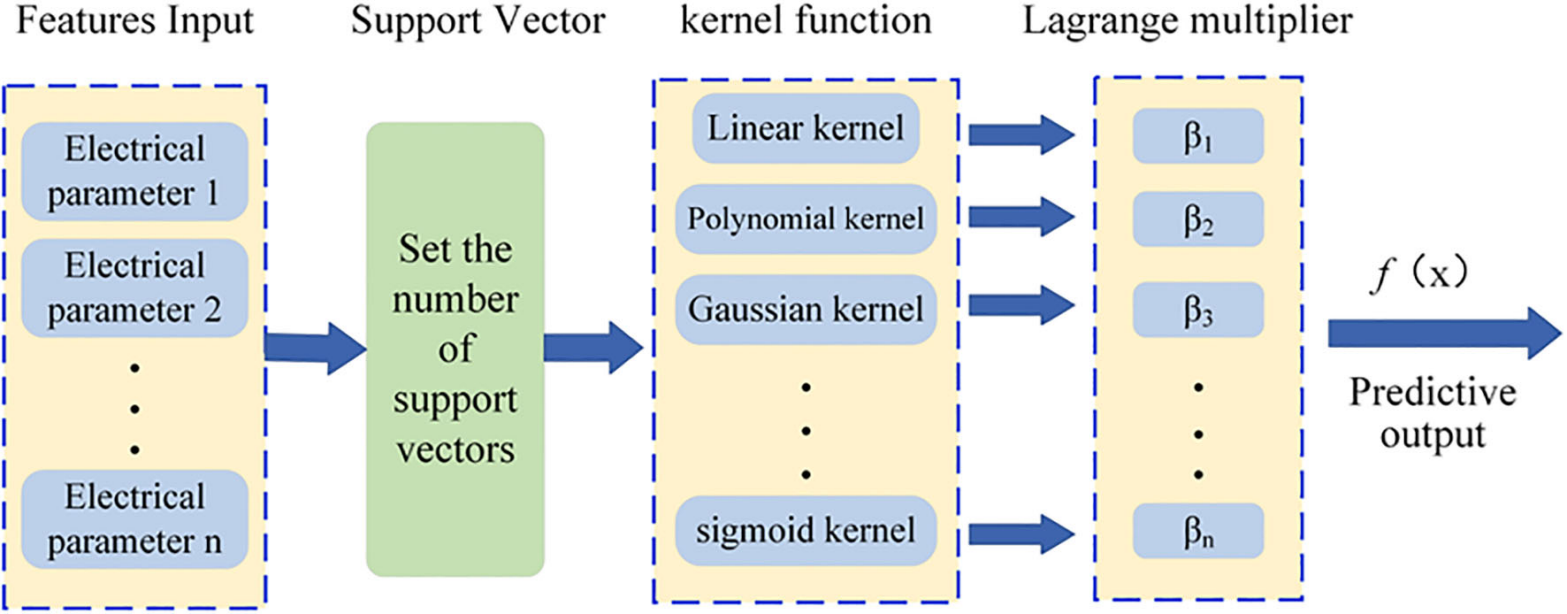
SVR model structure.


SVR is a machine learning algorithm for regression analysis, as shown in **Figure 5** based on the principles of SVM. It constructs a “margin band” around the linear function with a tolerance of ϵ, within which no loss is calculated for samples. Thus, only support vectors influence the function model. The optimal model is derived by minimizing total loss and maximizing the margin. The output function of the model, denoted as *f*(*x*), is illustrated in Equation 10.

(10) $$f(x)=\sum_{i=1}^{n}(\beta_{i}-\beta i^{*})\cdot k(x_{i},x)+b$$

Here, $\beta_{i}$ denotes the Lagrange multiplie, k represents the kernel function and b corresponds to the bias term.

In the SVR model, hyperparameter optimization is performed via cross-validation to determine the optimal values of ϵ, the kernel function, and the regularization parameter (C). The value of ϵ typically falls within the range [0, 1]. In this study, a grid search method is employed to select ϵ from the set [0.01, 0.1, 0.5, 1], and cross-validation is used to compare the performance of different ϵ values on the validation set to identify the optimal one. Commonly used kernel functions include the linear kernel, polynomial kernel, and Gaussian (RBF) kernel. The optimal kernel function is selected by comparing their performance on the validation set through cross-validation. The regularization parameter C, which controls the model complexity and the penalty for misclassified samples, is also optimized via grid search over the values [0.1, 1, 10, 100]. The hyperparameter configurations differ between the PCC-SVR and LSTMAE-SVR models, as detailed in **Tables 2**, **3**.

**Table 3:** The primary parameter configuration for PCC-SVR, PCC-XGBoost, and PCC-MLP.

Parameter settings		
Model name	Key parameters	Parameter settings
PCC-SVR	Kernel	rbf
	Epsilon(*ϵ*)	0.1
	Regularization parameter (C)	1
PCC-XGBoost	objective	reg:squarederror
	Base Learner	gbtree
	n_estimators	200
	max_depth	5
	min_child_weight	1
	gamma	1
	subsample	0.8
	colsample_bytree	1
	Learning Rate	0.01
PCC-MLP	Number of Hidden Layers	1
	Learning Rate	0.01
	batch_size	32
	epochs	200
	Activation Function	Sigmoid

#### 2.4.2 MLP model

**Figure 6:**
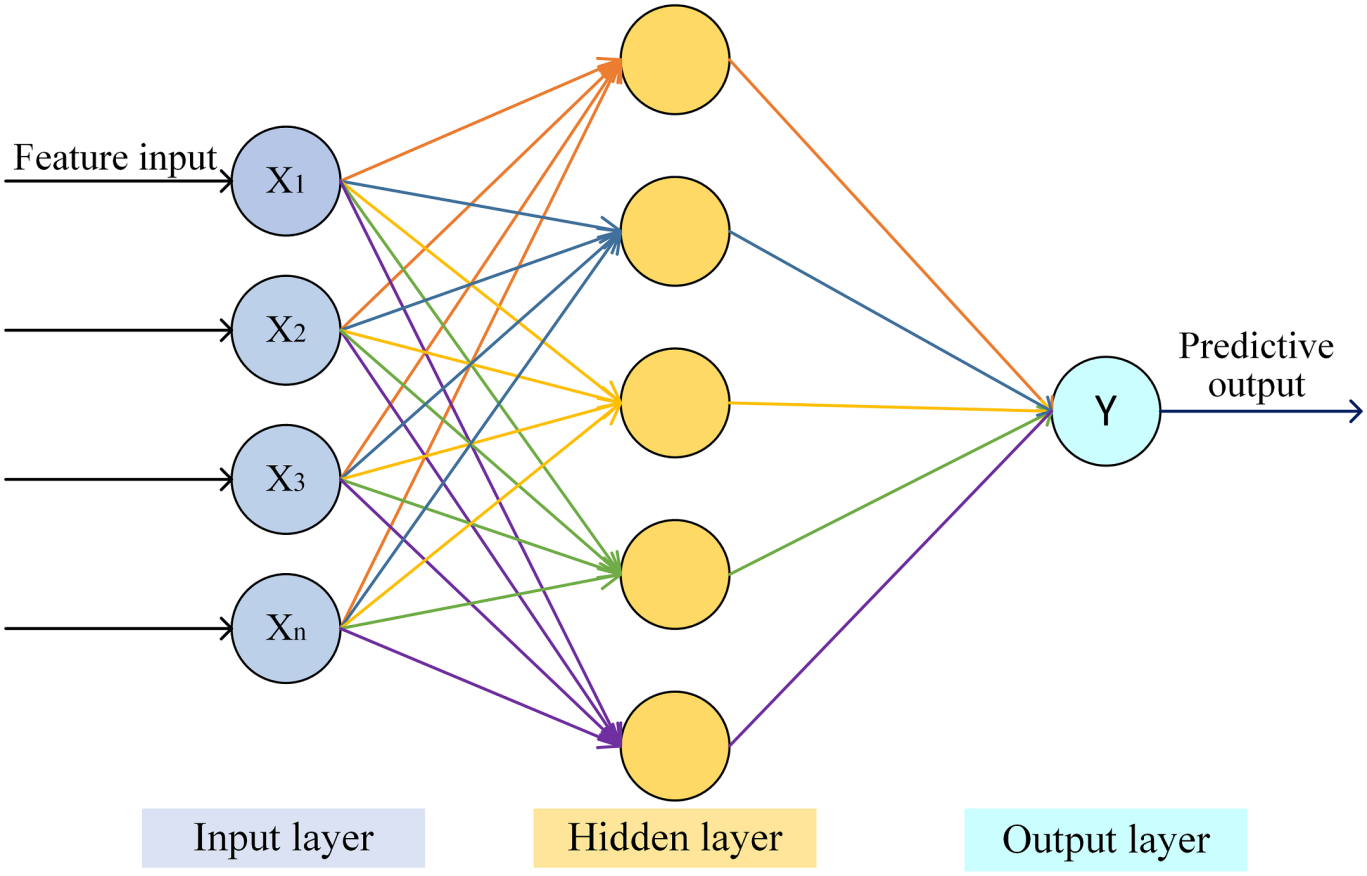
MLP model structure.

The MLP, also known as an Artificial Neural Network (ANN), consists of multiple layers including input and output layers, with one or more hidden layers in between. The simplest MLP has a single hidden layer, forming a three-layer structure. A simple MLP structure is depicted in **Figure 6**.

In the MLP model, the number of neurons in the input and output layers corresponds to the dimensionality of the input features and output targets, respectively. The hidden layer(s), situated between the input and output layers, serve as the core component for feature extraction and transformation. The network may contain one or multiple hidden layers, with each neuron fully connected to all neurons in the preceding layer. The prediction output $\hat{y}\;$ is computed as shown in Equation 11.


(11) $$\hat{y}=f(w^{\left(2\right)}\cdot \sigma (w^{\left(1\right)}\cdot x+b^{\left(1\right)})+b^{\left(2\right)})$$

In this formulation, $w^{\left(1\right)}\;$ denotes the weight matrix connecting the input layer to the hidden layer, while $b^{\left(1\right)}$ represents the bias vector of the hidden layer. Similarly, $w^{\left(2\right)}$ corresponds to the weight matrix between the hidden layer and the output layer, and $b^{\left(2\right)}$ indicates the bias vector of the output layer. The function σ refers to the activation function, and f designates the activation function applied in the output layer.

The MLP model primarily consists of an input layer, hidden layers, and an output layer. Key architectural hyperparameters to be defined include the number of layers and the number of neurons in each layer. In this study, the input layer is set to 10 neurons, one hidden layer with 16 neurons is used, while the output layer contains 1 neuron for the PCC-MLP model and 4 neurons for the LSTMAE-MLP model. Hyperparameters optimized during training include the learning rate, batch size, and number of epochs. The Adam optimizer is employed with an initial learning rate of 0.01. The batch size is set to 32 based on memory constraints, and training epochs are determined with the application of L1 regularization to prevent overfitting. The specific hyperparameter configurations for the MLP models are provided in **Tables 2** and **3**.

#### 2.4.3 XGBoost regression model

**Figure 7:**
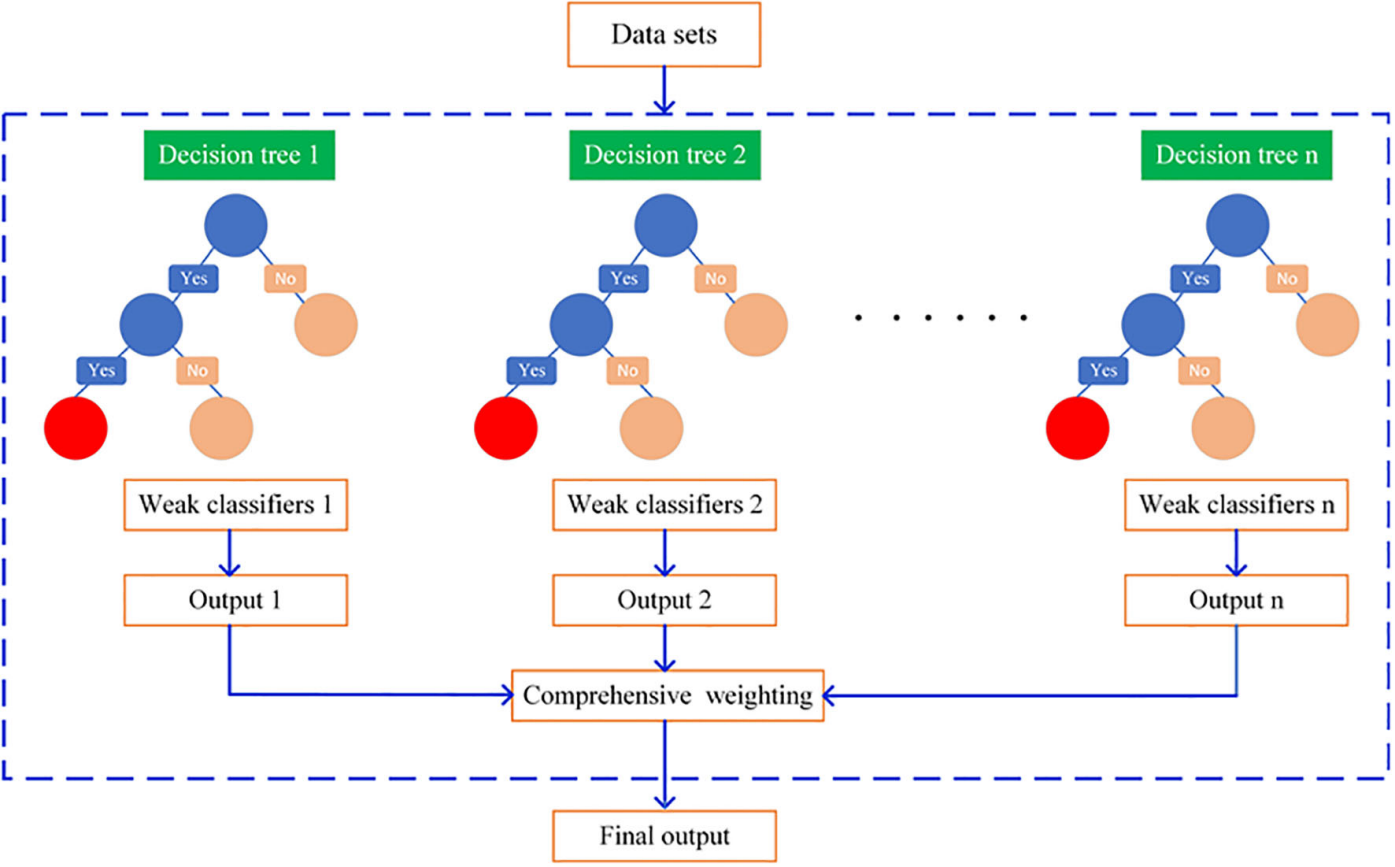
XGBoost model structure.


The Gradient Boosting Decision Tree (GBDT) algorithm employs gradient descent to construct new trees based on all previously developed trees, aiming to minimize the objective function as much as possible. The XGBoost algorithm is an enhancement built upon GBDT and is extensively utilized for both classification and regression tasks. As shown in **Figure 7**, by incorporating regression trees, the model continuously performs feature splitting learning at each internal node, generating new Classification and Regression Trees (CART), fitting the residuals from previous models, and recording the number of trees in the training model. The weights of the results corresponding to each tree are then combined to produce the final prediction values. The weights of the results corresponding to each tree are then combined to produce the final prediction values. The predicted value of the model is calculated as shown in Equation 12:

(12) $${\hat{y}}_{i}^{\left(t\right)}={\hat{y}}_{i}^{\left(t-1\right)}+\eta f_{t}(x_{i})$$

Here, ${\hat{y}}_{i}^{\left(t\right)}\;$ denotes the final predicted value after incorporating the t-th tree. ${\hat{y}}_{i}^{\left(t-1\right)}$ represents the cumulative prediction from the previous *t−*1 trees. The parameter η refers to the learning rate, which mitigates the influence of individual trees to prevent overfitting. The function $f_{t}(x_{i})$ corresponds to the predictive output of the t-th tree for the sample $x_{i}$ .

In the XGBoost model, hyperparameter optimization is performed via cross-validation to identify the optimal configuration, including the objective function, booster type, tree-specific hyperparameters (n_estimators, max_depth, min_child_weight, gamma, subsample, and colsample_bytree), and the learning rate. The objective function defines the learning objective of the model; in this study, the mean squared error is adopted for regression tasks. Among the available booster types—gbtree, gbtlinear, and dart—the optimal one is selected based on cross-validated performance on the validation set. For tree-specific hyperparameters, a grid search strategy is applied to determine their optimal values. It should be noted that the hyperparameter settings differ between the PCC-XGBoost and LSTMAE-XGBoost models, as summarized in **Tables 2**, **3**.

### 2.5 Software environment, data set partitioning, and model evaluation

The data processing environment for this paper is as follows: a computer equipped with an Intel(R) Core(TM) i7-10210U CPU and 8GB RAM; the machine learning environment includes Anaconda 23.1.0, Python 3.7.11, TensorFlow 2.0.0, and PyCharm.

Data sets were divided in an 8:2 ratio, with 235 data sets used for model training and 30 data sets for model testing and validation. Validation was conducted using three-fold cross-validation, with 29 data sets reserved for model testing. To evaluate the predictive performance of the model, this paper employs three commonly used metrics in machine learning: the determination coefficient (R^2^), Mean Absolute Error (MAE), and Root Mean Squared Error (RMSE). The calculation formula is shown in Equations 13–15. A higher R^2^ value, approaching 1, indicates better model fitting, while a lower RMSE value suggests improved model prediction accuracy. The mathematical expressions for these metrics are as follows:


(13) $$R^{2}=1-\frac{\sum_{i}{\left({\hat{y}}_{i}-y_{i}\right)}^{2}}{\sum_{i}{\left({\bar{y}}_{i}-y_{i}\right)}^{2}}$$

(14) $$MAE=\frac{1}{n}\sum_{i=1}^{n}|{\hat{y}}_{i}-y_{i}|$$

(15) $$RMSE=\sqrt{\frac{1}{n}\sum_{i=1}^{n}{\left(y_{i}-{\hat{y}}_{i}\right)}^{2}}$$

Here, n represents the number of samples, $y_{i}$ denotes the actual value the i th sample, ${\hat{y}}_{i}$ denotes the predicted value for the i th sample, and ${\bar{y}}_{i}$ denotes the mean of actual values.

## 3 Results

### 3.1 Correlation between electrical parameters and tomato quality indicators

Using the PCC method, a heatmap of the correlation between electrical parameters and four indicators was generated. The numbers in the image represent the values of the correlation coefficient r. The darker the color in the heatmap, the greater the correlation between the two observed values.

**Figure 8:**
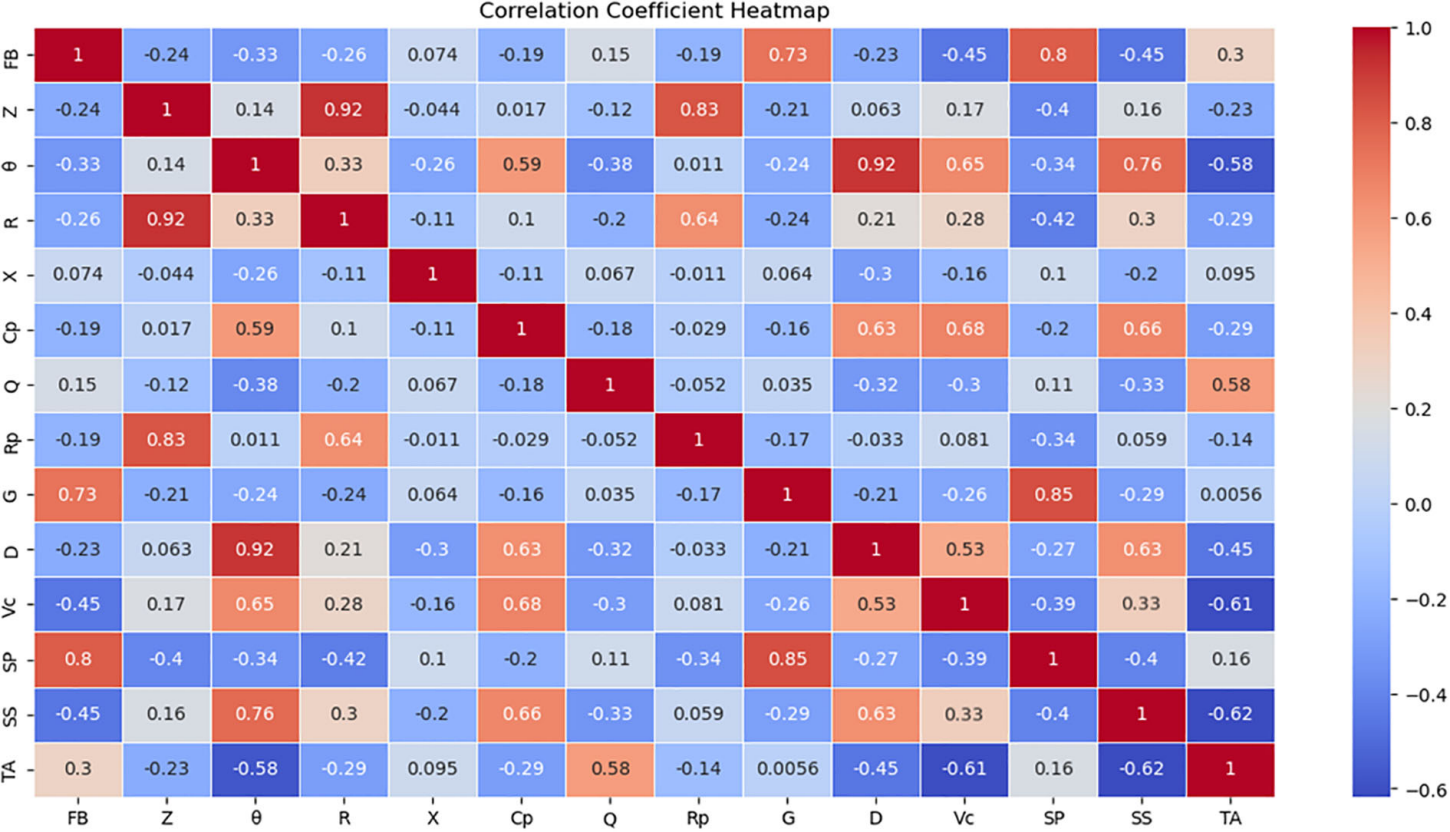
Correlation heatmap. In this figure, FB, SP, and SS represent Frequency Band, Soluble Protein, and Soluble Sugars, respectively.

As shown in **Figure 8**, there exists a certain correlation between vitamin C (VC), soluble protein, soluble sugars, titratable acidity (TA), and the electrical parameters. Parameters with higher correlation levels were selected for model construction. VC content demonstrated moderate positive correlations with capacitance (Cp, r=0.68), phase angle (θ, r=0.65), and dissipation factor (D, r=0.53). The variation in VC content is closely associated with the integrity of cellular structure, while parameters such as capacitance (Cp) and phase angle (θ) effectively capture these cellular-level changes. Consequently, Cp, θ, and D were selected as input features for predicting VC content using PCC-SVR, MLP, and XGBoost models.Soluble protein content showed a significant positive correlation with conductance (G). As the soluble protein content increases, it effectively enhances the internal ‘electrical conductivity’ of the tomato fruit, facilitating easier passage of electrical current, which manifests as an elevation in the G-value. Concurrently, a positive correlation was observed with frequency band variation indicators. Consequently, G and the Frequency Band parameter were selected as input features for predicting soluble protein content using the PCC-SVR, MLP, and XGBoost models. Soluble sugar content demonstrated moderate positive correlations with phase angle (θ, r=0.76), parallel capacitance (Cp, r=0.66), and dissipation factor (D, r=0.63). These parameters exhibit high sensitivity to variations in internal sugar content and were consequently selected as input features for predicting soluble sugar concentration using PCC-SVR, MLP, and XGBoost models. Titratable acid showed a moderate correlation with θ (r = -0.58) and Q (r = 0.58). An increase in acidity enhances the resistive pathway by increasing ionic conductivity, reduces the capacitive response (θ decreases), and simultaneously optimizes the energy transmission efficiency (Q increases). Therefore, θ and Q were selected as input features for predicting titratable acid content using PCC-SVR, MLP, and XGBoost models.

### 3.2 Prediction results based on PCC method

Using feature variables reduced via PCC as inputs and the quality indices of tomatoes—vitamin C, soluble sugars, soluble proteins, and titratable acidity—as outputs, predictions were analyzed using three machine learning regression algorithms: SVR, XGBoost, and MLP.

As shown in **Table 4**, three models are evaluated using RMSE, MAE, and R². In the context of the application scenario presented in this paper, these four indicators are specific numerical values. The primary objective is to assess the accuracy of prediction errors. Given that the dataset used in this study is relatively homogeneous with few outliers, RMSE is selected as the primary evaluation metric for the models. Secondary attention is given to the goodness of fit of the models, with R² serving as the second evaluation criterion. Lastly, considering the potential outliers in the overall dataset, MAE is listed as the third evaluation metric.

**Table 4:** Evaluation results for hybrid models.

Evaluation results of different models based on LSTMAE							
Prediction indices	Model name	Training set			Test set		
		RMSE	MAE	R^2^	RMSE	MAE	R^2^
Vitamin C (mg/100g)	LSTMAE-SVR	0.872	0.504	0.879	0.930	0.633	0.732
	**LSTMAE-XGBoost**	**1.039**	**0.271**	**0.881**	**1.041**	**0.524**	**0.805**
	LSTMAE-MLP	0.943	0.547	0.896	0.811	0.313	0.726
Soluble Sugars (%)	LSTMAE-SVR	1.614	1.273	0.690	1.613	1.277	0.680
	**LSTMAE-XGBoost**	**0.321**	**0.190**	**0.955**	**0.473**	**0.215**	**0.945**
	LSTMAE-MLP	0.882	0.678	0.678	0.971	0.890	0.874
Soluble Proteins (mg/g)	LSTMAE-SVR	0.306	0.183	0.790	0.313	0.211	0.760
	**LSTMAE-XGBoost**	**0.249**	**0.141**	**0.919**	**0.294**	**0.191**	**0.838**
	LSTMAE-MLP	0.360	0.273	0.704	0.452	0.370	0.680
Titratable Acidity (mmol/100g)	LSTMAE-SVR	0.037	0.019	0.890	0.068	0.025	0.640
	**LSTMAE-XGBoost**	**0.038**	**0.013**	**0.956**	**0.041**	**0.018**	**0.845**
	LSTMAE-MLP	0.045	0.020	0.789	0.048	0.021	0.753

The units of RMSE and MAE are identical to those of the original predicted variables. Bold values represent the group with the best performance in model comparison.

The features with high relevance were extracted as input variables using the PCC method. After normalization, Cp, θ, and D were utilized as inputs, with Vc content serving as the output. After adjusting the model parameters, the results are presented in **Table 2**. The PCC-XGBoost model exhibited the best prediction performance for Vc content. Although its R² value on the test set was slightly lower than that of the PCC-SVR model, it had a lower Mean Absolute Error (MAE), indicating a smaller average discrepancy between the predicted and actual values.

**Figure 9:**
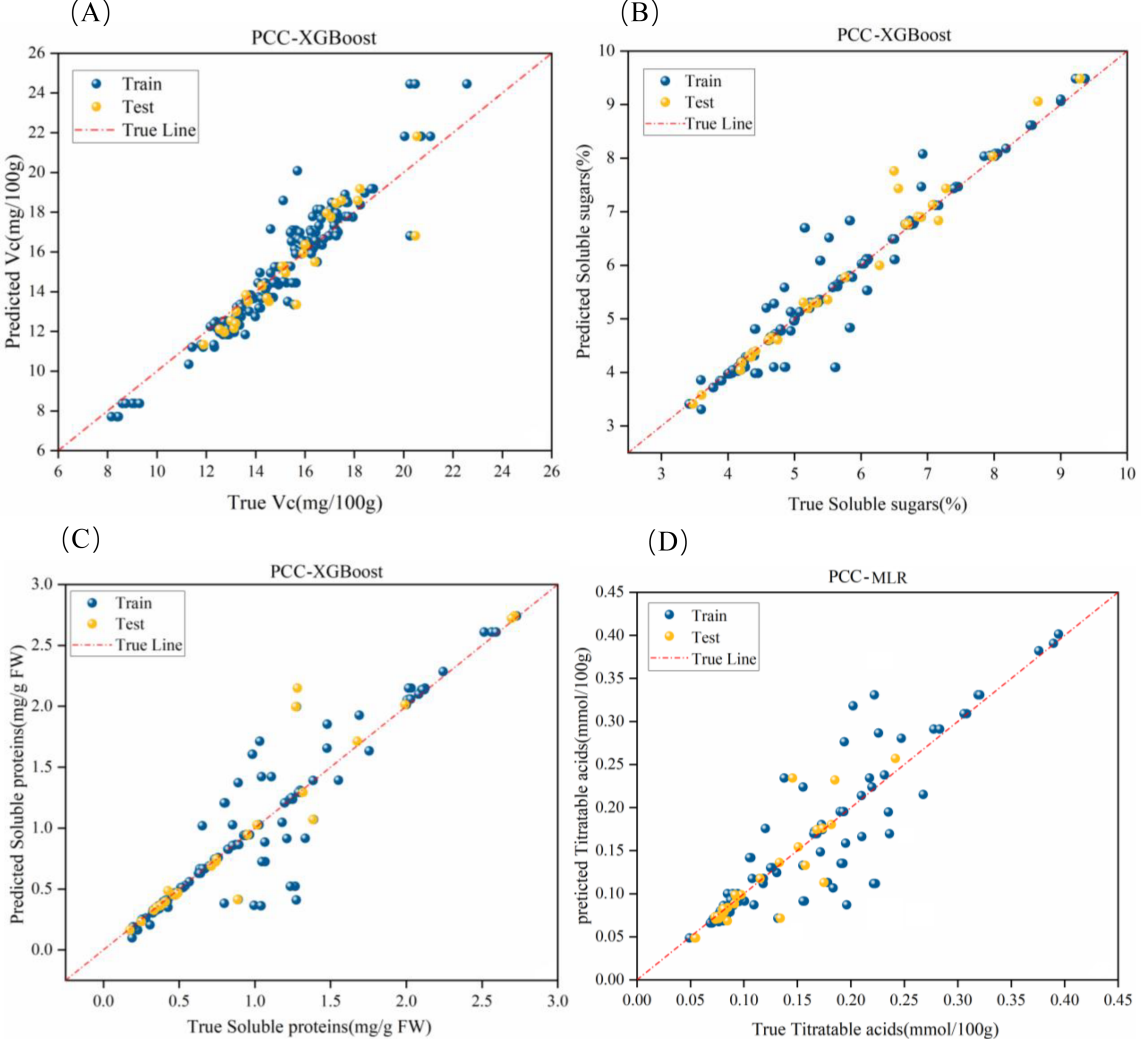
Optimal fitting effects of PCC-based hybrid models. **(a-d)** represent the fitting effects for vitamin C, soluble sugars, soluble proteins, and titratable acidity, respectively.

For Soluble Sugars, Cp, D, and θ were used as inputs, with Soluble Sugars as the output. After adjusting the model, PCC-XGBoost was identified as the optimal model, achieving an R² of 0.814 on the test set, indicating good prediction performance. However, as shown in **Figure 9b**, there were still considerable deviations in the prediction within the 5-8% range, suggesting further model optimization is required.

Regarding the Soluble Proteins indicator, Frequency band and G were used as inputs, with Soluble Proteins as the output. After model adjustment, PCC-XGBoost was determined to be the optimal model due to its lower Root Mean Square Error (RMSE) in the test set. However, as illustrated in **Figure 9c**, there were still significant deviations in the prediction of soluble protein content within the 0.1-0.28 mg/gFW range, indicating a need for further model optimization.

For titratable acidity, Q and θ were used as inputs, with titratable acidity content as the output. After adjusting the model, the PCC-MLP model was found to have better prediction performance, with an R² of 0.75 in the test set. This result may be at-tributed to the complexity of parameter tuning in the XGBoost model compared to MLP, especially with a smaller dataset, making it challenging to adjust the optimal combination of parameters. This led to the poorer performance of PCC-XGBoost in predicting titratable acidity. As shown in **Figure 9d**, the model exhibited larger errors in the 0.15-0.25 mmol/100g range.

### 3.3 Predictive outcomes based on the LSTMAE model

When combined with prediction models, LSTMAE eliminates the need for manual feature selection using PCC models. Simply import the electrical parameter data into the model, set the data interval to 5 according to the electrical parameter frequency band, and configure other parameters.

The model can then automatically learn features, which are subsequently fed into regression models such as SVR, XGBoost, and MLP for prediction. This entire process requires no human intervention, enabling end-to-end learning. Leveraging the characteristics of end-to-end learning, the output of the prediction model is set to 4, meaning that a single model can simultaneously predict four indicators, thereby reducing model redundancy.

**Figure 10:**
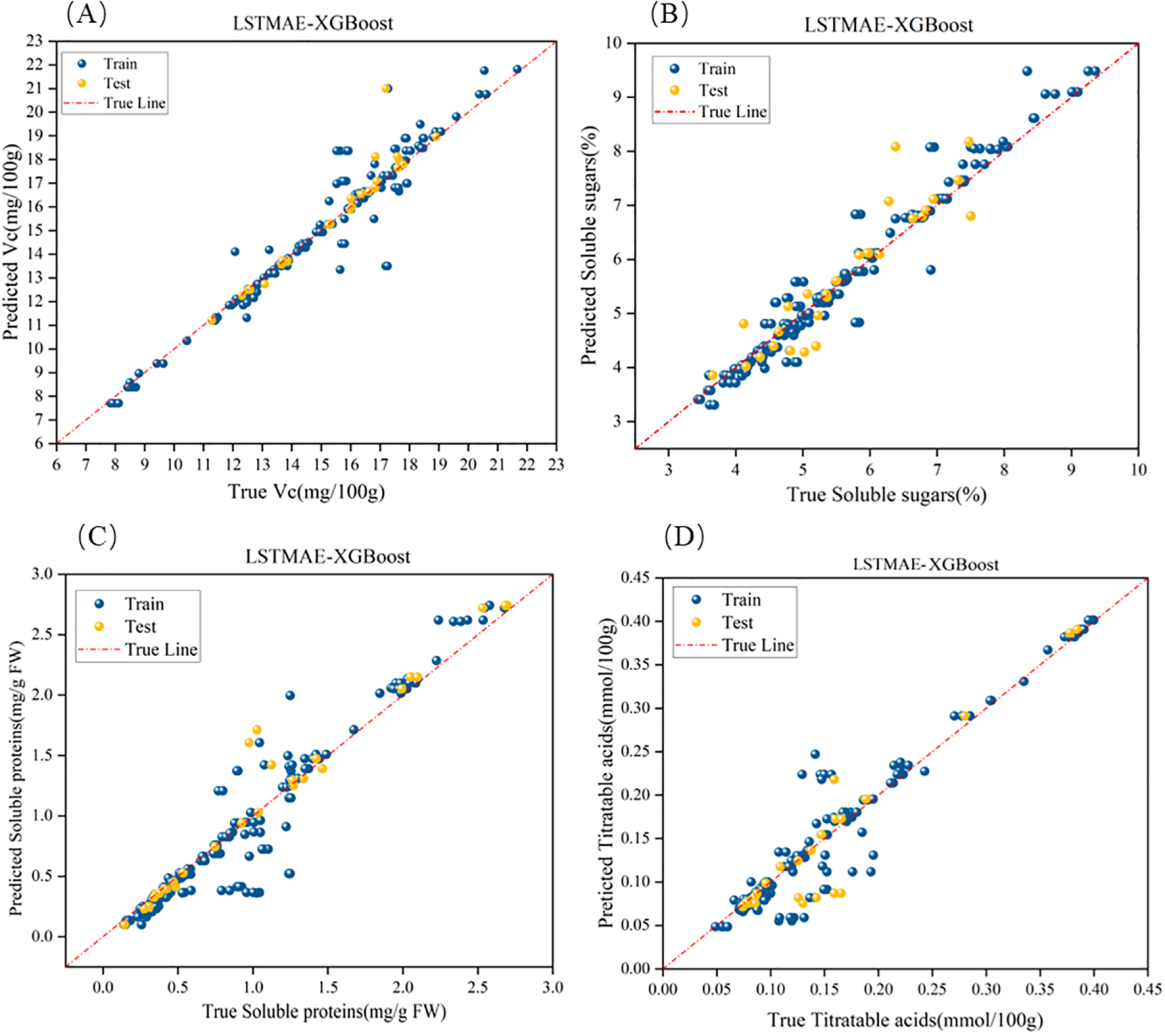
Optimal fitting effects of the hybrid LSTMAE model. **(a**–**d)** represent the fitting performance for Vitamin C, soluble sugars, soluble proteins, and titratable acids, respectively.

The results presented in **Table 5** and **Figure 10** demonstrate that the LSTMAE-XGBoost model outperforms the other two models in terms of several metrics. Specifically, its RMSE is lower than that of the other two models, indicating a smaller deviation between the predicted values and the actual values compared to the other models. Additionally, the R^2^ value of the LSTMAE-XGBoost model is higher, suggesting that its overall data predictions are closer to the fitted regression line than those of the other models. Except for the Vc indicator, the MAE of all indicators for the LSTMAE-XGBoost model is also lower than that of the other two models, which implies a reduction in the average prediction error when using this model.

**Table 5:** Evaluation results of different models based on LSTMAE.

Evaluation results for hybrid models							
Prediction indices	Model name	Training set			Test set		
		RMSE	MAE	R^2^	RMSE	MAE	R^2^
Vitamin C (mg/100g)	PCC-SVR	2.186	2.072	0.753	2. 809	2. 689	0.680
	PCC-XGBoost	**1.013**	**0.713**	**0.786**	**1.105**	**0.746**	**0.662**
	PCC-MLP	1.636	1.240	0.673	2.012	1.733	0.527
Soluble Sugars (%)	PCC-SVR	0.974	0.726	0.614	1.008	0.749	0.604
	PCC-XGBoost	**0.309**	**0.155**	**0.858**	**0.386**	**0.176**	**0.814**
	PCC-MLP	0.908	0.740	0.655	0.826	0.743	0.659
Soluble Proteins (mg/g)	PCC-SVR	0.368	0.220	0.747	0.432	0.358	0.736
	PCC-XGBoost	**0.223**	**0.09**	**0.819**	**0.384**	**0.160**	**0.760**
	PCC-MLP	0.242	0.126	0.894	0.465	0.183	0.855
Titratable Acidity (mmol/100g)	PCC-SVR	0.074	0.043	0.680	0.081	0.062	0.570
	PCC-XGBoost	0.062	0.023	0.744	0.068	0.025	0.616
	PCC-MLP	**0.045**	**0.019**	**0.789**	**0.058**	**0.027**	**0.750**

The units of RMSE and MAE are identical to those of the original predicted variables. Bold values represent the group with the best performance in model comparison.

As can be seen from **Table 5** and **Figure 10**, LSTMAE-XGBoost outperforms the other two models in predicting all four indicators. This model is capable of extracting complex relationships, time series features, and non-Gaussian features from electrical parameter data, demonstrating exceptional prediction performance.

### 3.4 Model comparison analysis

Overall, the results indicate that the LSTMAE model is an effective method for feature extraction, achieving superior outcomes compared to traditional PCC-based feature extraction. It is particularly useful for feature extraction and enhancement in tomato samples. Experimental findings suggest that by measuring the electrical properties of tomatoes, it is possible to effectively predict internal indices such as vitamin C, soluble sugars, soluble proteins, titratable acidity, and nitrate content, with the model demonstrating robust performance in predicting these internal indices.

Whether using the LSTMAE or PCC model to extract feature variables, the XGBoost model demonstrates better fitting performance, followed by the MLP model. The SVR model exhibits poorer fitting performance compared to the first two models. When comparing the optimal model for each indicator, the combined model based on LSTMAE exhibits reduced errors on both the training and validation sets, with improvements in the coefficient of determination R². Comparisons of R² and RMSE for the test set are shown in **Table 6**.

**Table 6:** Comparative effects of feature extraction methods: LSTMAE vs. PCC.

Comparative effects of feature extraction methods: LSTMAE vs PCC				
Prediction indicators	Evaluation metrics	PCC-XGBoost	LSTMAE-XGBoost	Value of variation
Vitamin C	R^2^	0.662	0.805	+14.3%
	RMSE	1.105	1.041	-0.064
Soluble protein	R^2^	0.814	0.945	+13.1%
	RMSE	0.386	0.473	+0.087
Soluble sugars	R^2^	0.760	0.838	+7.8%
	RMSE	0.384	0.294	-0.09
Titratable Acids	R^2^	0.750	0.845	+9.5%
	RMSE	0.058	0.041	-0.017

## 4 Discussion

This study explores the feasibility of constructing a non-destructive detection model for tomato internal quality indicators based on machine learning and electrical parameters. Utilizing electrical parameter data and quality indicators of tomatoes, various machine learning models with different feature extraction methods were established and compared.

The first method involves the manual selection of feature variables that demonstrate high correlation with quality indicators by comparing Pearson correlation coefficients. Upon comparison, it was observed that Cp, θ, and D exhibited strong correlation with the vitamin C indicator, aligning with the findings reported by Ye, L et al. in their research on kiwifruit (Tang et al., 2017). G exhibits a high correlation with soluble sugars, while θ, Cp, and D demonstrate strong correlations with soluble protein indicators. The majority of studies have primarily focused on exploring the relationship between soluble solids content (SSC) and electrical parameters, noting that SSC comprises both soluble sugars and soluble proteins. In the research conducted by Ye, L et al., SSC was found to correlate with Rp, Q, D, and G. Similarly, in the study by Tang,Y.R et al., a significant correlation between SSC and parameters such as Cp, Lp, and D was observed in Korla pear (Tang et al., 2012). The parameters θ and Q exhibit a strong correlation with the titratable acidity (TA) index, a finding that aligns with the research conducted by Yan, T. et al (Shao et al., 2010), on TA in kiwifruit. Conversely, in studies focusing on apples and persimmons, the electrical parameters Z and Rs show a higher correlation with TA. These two parameters can be employed to develop a regression prediction model (An et al., 2013; Mitiche et al., 2021). Differences in quality indicators and highly correlated electrical parameters arise due to variations in measurement instruments, methodologies, and types of fruits and vegetables being measured. In this study, we utilized selected features as inputs and the corresponding quality indicators as outputs to establish a machine learning model for non-destructive testing.

Due to the variation in features selected by the PCC method for four different indicators, a prediction model is required for each indicator. We utilized SVR, MLP, and XGBoost to construct models for these four indicators. By comparing evaluation metrics, it was found that the XGBoost model outperformed the SVR and MLP models for Vc, soluble sugars, and soluble proteins. However, for titratable acidity, the MLP model was superior to both the XGBoost and SVR models. This result may be attributed to the better parameter adjustment of the MLP model when predicting the titratable acidity indicator. Nevertheless, the advantages of the XGBoost model cannot be denied. The superior performance of the XGBoost model in this study stems from the inherent alignment between its algorithmic design and the characteristics of both the data and the task: (1) It automatically captures complex nonlinear relationships between electrical parameters and quality indicators, whereas SVR and MLP either struggle with such complexity or require more intricate hyperparameter tuning to achieve comparable performance; (2) Built-in regularization mechanisms effectively mitigate overfitting, which is particularly critical given the limited sample size of this study;(3) Tree-based models like XGBoost possess a natural aptitude for handling structured tabular data; (4) Its gradient boosting optimization process exhibits greater efficiency and stability compared to the backpropagation used in MLP and the quadratic programming required for SVR. Thus, the outstanding results achieved by XGBoost reflect intrinsic advantages of its algorithm design over other models within the context of this application.

The second approach employs automatic feature extraction to construct a non-destructive testing model. LSTMAE integrates the temporal data analysis capabilities of LSTM with the feature learning and dimensionality reduction abilities of autoencoders, thereby enabling efficient processing of multidimensional time series data (Luo et al., 2025). The XGBoost model employs regularization during the training process to control model complexity, thereby preventing overfitting and enhancing model robust-ness (Cheng et al., 2025). The superiority of the XGBoost model has also been noted in the study by Cheng, T. et al (Morales-Hernández et al., 2023). Through comparison, it was found that the method utilizing automatic feature extraction outperformed the manual feature extraction method, with improvements observed in the determination coefficient R^2^ of the models. When features were manually extracted, different electrical parameters were selected for each indicator, necessitating the construction of a separate model for each indicator. In contrast, automatic feature extraction allowed for the simultaneous consideration of four pre-diction indicators as outputs, enabling the establishment of a multi-output model that enhanced model efficiency. Therefore, this study concludes that LSTMAE-XGBoost represents an effective model for non-destructive testing of tomato indicators, providing new insights for subsequent nondestructive testing models that combine electrical properties with machine learning.

In the research process of this paper, although improvements were made to the feature extraction method, the adjustment of model parameters was still based on experience and experimental results. In the field of deep learning optimization, various hyperparameter optimization techniques exist, such as grid search, random search, and dynamic resource allocation algorithms (Mitiche et al., 2021). Future research work will incorporate other parameter optimization methods to further refine the model.

## 5 Conclusions

The physicochemical indices within tomatoes are crucial determinants of tomato quality and flavor, and fruit quality inspection techniques and assessments are key to advancing the industry, playing a significant role in tomato grading, classification, and providing clear choices for consumers. This paper utilized two feature extraction methods (LSTMAE and PCC) and three regression prediction models (SVR, MLP, and XGboost) to predict four quality indices of tomatoes. The main findings are as follows:

1. By comparison, it was found that using the LSTMAE model for input feature extraction outperformed PCC. The coefficients of determination (R²) for vitamin C, soluble sugar, soluble protein, and titratable acidity improved by 14.3%, 13.1%, 7.8%, and 9.5%, respectively.
2. When using PCC for feature extraction and recombining the extracted features as input for three regression models—SVR, MLP, and XGBoost—PCC-XGBoost out-performed the other two models in predicting vitamin C, soluble sugar, and soluble protein indicators. However, for the prediction of titratable acidity, the PCC-MLP model provided better results.
3. By utilizing LSTMAE for automatic feature learning, LSTMAE-XGBoost out-performed the other two models in predicting vitamin C, soluble sugar, and soluble protein indicators. Additionally, the LSTMAE-XGBoost model can simultaneously predict all four indicators, resulting in superior model efficiency compared to traditional machine learning models.

These results provide technical and theoretical support for non-destructive detection of internal quality indices in tomatoes and can offer insights for traditional operational models in the tomato industry, promoting the advancement of non-destructive testing techniques and providing guidance for rapid and non-destructive inspection of fruit quality.

## Data Availability

The raw data supporting the conclusions of this article will be made available by the authors, without undue reservation.
